# Three-dimensional dominant frequency mapping using autoregressive spectral analysis of atrial electrograms of patients in persistent atrial fibrillation

**DOI:** 10.1186/s12938-016-0143-8

**Published:** 2016-03-08

**Authors:** João L. Salinet, Nicholas Masca, Peter J. Stafford, G. André Ng, Fernando S. Schlindwein

**Affiliations:** Biomedical Engineering, Modelling and Applied Social Sciences Centre, Federal ABC University, Bloco Delta, Sala 335 - Rua Arcturus, 03 - Jardim Antares, São Bernardo do Campo, SP CEP 09606-070 Brazil; Department of Engineering, University of Leicester, Leicester, UK; Department of Cardiovascular Sciences, University of Leicester, Glenfield Hospital, Leicester, UK; Leicester NIHR Biomedical Research Unit in Cardiovascular Disease, Glenfield Hospital, Leicester, UK

**Keywords:** Autoregressive model, Atrial fibrillation, Unipolar electrograms, Noncontact mapping, Dominant frequency mapping

## Abstract

**Background:**

Areas with high frequency activity within the atrium are thought to be ‘drivers’ of the rhythm in patients with atrial fibrillation (AF) and ablation of these areas seems to be an effective therapy in eliminating DF gradient and restoring sinus rhythm. Clinical groups have applied the traditional FFT-based approach to generate the three-dimensional dominant frequency (3D DF) maps during electrophysiology (EP) procedures but literature is restricted on using alternative spectral estimation techniques that can have a better frequency resolution that FFT-based spectral estimation.

**Methods:**

Autoregressive (AR) model-based spectral estimation techniques, with emphasis on selection of appropriate sampling rate and AR model order, were implemented to generate high-density 3D DF maps of atrial electrograms (AEGs) in persistent atrial fibrillation (persAF). For each patient, 2048 simultaneous AEGs were recorded for 20.478 s-long segments in the left atrium (LA) and exported for analysis, together with their anatomical locations. After the DFs were identified using AR-based spectral estimation, they were colour coded to produce sequential 3D DF maps. These maps were systematically compared with maps found using the Fourier-based approach.

**Results:**

3D DF maps can be obtained using AR-based spectral estimation after AEGs downsampling (DS) and the resulting maps are very similar to those obtained using FFT-based spectral estimation (mean 90.23 %). There were no significant differences between AR techniques (p = 0.62). The processing time for AR-based approach was considerably shorter (from 5.44 to 5.05 s) when lower sampling frequencies and model order values were used. Higher levels of DS presented higher rates of DF agreement (sampling frequency of 37.5 Hz).

**Conclusion:**

We have demonstrated the feasibility of using AR spectral estimation methods for producing 3D DF maps and characterised their differences to the maps produced using the FFT technique, offering an alternative approach for 3D DF computation in human persAF studies.

## Background

Atrial fibrillation (AF) is the commonest heart rhythm disturbance seen in clinical practice, affecting almost 1 % of the worldwide population. It is more prevalent in older patients, affecting over 10 % of those above 80 years old [1]. AF increases the risk of stroke fivefold and the risk of heart failure and mortality [2]. It is reason for frequent medical appointments and admissions, contributing to elevated costs of health care [2]. Despite extensive research into the pathophysiology of AF, the mechanisms of its triggering and maintenance are still controversial and effective treatment is still elusive [3]. The success of using catheter ablation in paroxysmal AF patients has illustrated the usefulness of the technique [4], however the treatment of persistent AF (persAF) via ablation is still a challenge. Different techniques have been employed to improve ablation outcome in persAF [5] and it has been suggested that dominant frequency (DF), defined as the frequency of the highest peak of the AF frequency spectrum, could help identify targets for ablation [6]. Investigators using spectral analysis observed that the activation rates of localized endocardium areas were well correlated with DF [7]. It has been suggested that regions with highest DF may be responsible for the AF maintenance and hence should be targets for ablation [8]. This highlights the need for their accurate localization and a system with high-resolution simultaneous endocardial recordings [6, 9] enables accurate mapping and facilitates targeting of potential arrhythmic sites and circuits [9]. Spectral analysis of these electrograms may serve as a powerful tool for identifying AF candidates [6, 9].

The majority of the AF studies that studied the DF [10] were implemented using Fourier-based spectral analysis techniques. As the temporal behaviour of DF is not stable [11], it is desirable to track its trajectory over time using short segments of atrial electrograms for the spectral analysis. It is well known that Fourier-based spectral analysis suffers from low spectral resolution when the length of the time segment is short [12–14]. In these cases, AR-based spectral analysis might be an interesting alternative as it has superior spectral resolution [13].

Therefore, the objectives of this paper were (1) to generate 3D DF maps using different autoregressive (AR) spectral estimation methods [with emphasis on selection of appropriate sampling rate and AR model order to estimate the DF for each of those 2048 simultaneous unipolar noncontact intracardiac atrium electrograms (AEGs)], (2) to compare the DF between those AR spectral estimation techniques and (3) to compare them with the maps produced using Fourier-based approach [15].

## Methods

### Overview of noncontact mapping

Noncontact mapping (NCM) is performed with a multielectrode array catheter (MEA) introduced into the cardiac chamber to record endocardial electrical activity without touching the heart walls. This technology uses an array catheter with 64 electrodes and an analysis system (EnSite 3000, St. Jude Medical) that generates anatomic mapping and electrogram reconstruction. The simultaneous potentials are obtained using inverse solution mathematics and up to 3600 AEGs sampled at 1200 Hz are projected onto 3D representation of the cardiac chamber in real-time [16].

The technique of using NCM with the MEA has previously been described and validated in the context of sinus rhythm as well as arrhythmia in humans [17, 18]. Estimation of DF via spectral analysis from NCM has been shown to be well correlated (agreement in approximately 95 % of cases) with DF estimation via spectral analysis from contact mapping for both paroxysmal AF and persAF [17, 18] and can be used in simultaneous high density 3D DF maps as a tool to identify sites with high frequency electrical activity during AF [18].

### Data collection

An Ensite array balloon was introduced trans-septally into the LA of eight patients with persAF with no previous history of heart diseases (patient characteristics are summarised in Table 1). Patients were in AF and 2048 AEGs for 20.478 s-long segments were exported for analysis (3 × 6.826 s), together with their anatomical locations. The sampling frequency (Fs) was 1200 Hz. The length of the time window is such that it is long enough to allow the tracking of DF along time, as the time constant associated to the stability of DFs has been shown to be about 10 s [11]. The AEGs were high-pass filtered at 1 Hz and, apart from the built-in anti-aliasing filter, no further filtering or pre-processing prior spectral analysis was applied to the signals to preserve signal integrity and low frequency components [7]. Approval was obtained from the Local Ethics Committee for patients undergoing AF ablation including blood sampling and collection of electrical data and all procedures were carried out after informed consent.

**Table 1:** Clinical patients’ characteristics

	Patients (n = 8)
Mean age (years)	47 ± 4
History of persistent AF (months)	34 ± 9
Male (no.)	8
Hypertension (no.)	2
LV function (no.)	
EF ≥ 55 %	5
EF 45–54 %	2
EF 36–44 %	0
EF ≤ 35 %	1
LA Size (mm)	48 ± 2
On Amiodarone (no.)	3

*AF* indicates atrial fibrillation, *LV* left ventricular, *EF* ejection fraction, *LA* left atrial

### Spectral analysis

**Fig. 1:**
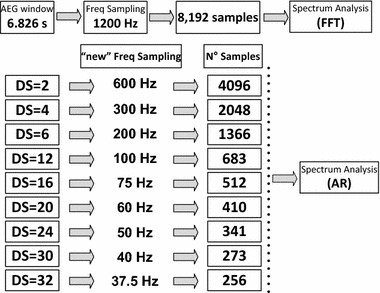
Illustration of the downsampling of the AEGs with nine different downsampling strategies: from 1200 down to 37.5 Hz prior to spectral analysis followed by its spectral analysis strategy

**Fig. 2:**
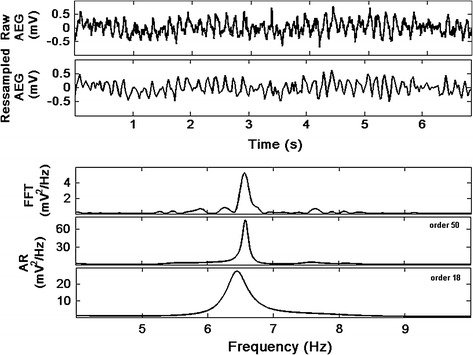
AEG (raw signal) sampled at 1200 Hz (*upper trace*) and corresponding signal after downsampling 32 times (Fs = 37.5 Hz). PSD estimation of the raw signal using FFT, followed by the PSD of the downsampled signal using AR Yule-Walker approach with model order p = 50 and p = 18

AR model-based spectrum analysis of the AEGs was performed after the signals were downsampled in the time domain with nine different sampling frequency values, from 600 Hz down to 37.5 Hz prior to spectral analysis (Fig. 1). The downsampling AEG process was performed by the function ‘resample’ of the Matlab 64-bit R2012a through the Signal Processing toolbox [version 6.17]. In this function an anti-aliasing (low-pass) linear-phase FIR filter is implemented with a Kaiser window to minimize the weighted, integrated squared error between the ideal piecewise linear function and the filter magnitude response. The method follows up sampling the output signal to then insert zeros. The resulting signal is filtered by a FIR and downsampled from 1200 to 37.5 Hz and illustrated on Fig. 1. An AEG recorded from the LA sampled originally at 1200 Hz is presented on Fig. 2. The respective signal is also displayed after a downsampling factor of 32 times (re-sampling frequency = 37.5 Hz) illustrating how much of the signal information is preserved.

After the re-sampling strategy, 3D DF maps with 2048 AEGs were produced for the different frequency sampling strategies to assess the behaviour of DF maps. DF was defined as the fundamental frequency with the highest power between 4 and 12 Hz [10] after the ventricular far field cancellation using a previously described algorithm [19]. The results obtained through the AR model-based methods were compared with DF maps recently described using the Fourier-based approach [15].

### Autoregressive model analysis

In the AR model approach [20–22], the signal *x*[*n*] is modelled as the output of an all-pole filter of model order *p* with coefficients *a*[*k*] for a white noise input driving sequence *w*[*n*] as follows.

1 $$ x[n] = w[n] - \sum\limits_{k = 1}^{p} {a[k]\;x[n - k]}. $$

The power spectral density (PSD) of an autoregressive process *P*_*AR*_ [*f*] is the continuous function of *f* as given by Eq. (2) [20–22],

2 $$ P_{AR} \left[ f \right] = \frac{{\sigma^{2} T}}{{\left| {1 + \sum\nolimits_{k = 1}^{p} {a_{{_{k} }}^{ - j2\pi fkT} } } \right|^{2} }} $$

where *σ*^*2*^ is the variance of the driving white noise *w*[*n*] and *T* is the sampling period.

To estimate the AR coefficients $\left\{ {a_{1} , \, a_{2} , \, a_{3} , \ldots , \, a_{p} , \, \sigma^{2} } \right\}$ a relationship between the AR parameters and the autocorrelation function that minimizes the residual error (the difference between *x*[*n*] and the output of the all-pole filter as given later by Eq. 5) has been derived and is known as the Yule-Walker equations (Eqs. 3 and 4 below). The derivation is shown by Kay and Marple in [20].

3 $$ R_{xx} \left( k \right) = - \sum\limits_{l = 1}^{p} {a_{m} \,\,R_{xx} \left( {k - m} \right) \, \,\,\,\,\,{\text{for }}k > 0} $$

4 $$ R_{xx} \left( k \right) = - \sum\limits_{l = 1}^{p} {a_{m} \,\,R_{xx} \left( { - m} \right) + \sigma^{2} \,\,\,\,\,{\text{for}}\,k = 0} $$

The verification that an autoregressive model can describe the AEG signals was performed by fitting an AR model to the AEG data and testing the residuals. The difference between the predicted and the true signal sequence values resulted in random residuals (uncorrelated in time) normally distributed. The autocorrelation function of the residuals fell inside the confidence interval of 95 % and was close to zero for all non-zero lags.

Different AR spectral estimation methods represent a trade-off between spectral estimation and computational efficiency [14, 20, 22]. Levinson Durbin Yule-Walker, Covariance, Modified Covariance and Burg).

#### Levinson-Durbin Yule-Walker method

The parameters of an AR process with zero mean and model order p using the Yule-Walker method with the Levinson-Durbin recursive algorithm are the solution of a set of linear equations which are obtained by the minimization of the estimate of the prediction error power (Eq. 5), with the extrapolation of the known estimated values (k) of the autocorrelation function (ACF) R_xx_ (Eq. 6). The algorithm has the advantage of being computationally efficient, requiring a number proportional to p^2^ mathematical operations and guarantees that the estimated poles lie within the unit circle.

5 $$ \sigma^{2} = \frac{1}{N}\sum\limits_{n = - \infty }^{\infty } {\left| {x\left[ n \right] + \sum\limits_{k = 1}^{p} {a[k]x[n - k]} } \right|}^{2} $$

6 $$ R_{xx\left[ k \right]} = \frac{1}{N}\sum\limits_{n = 0}^{N - k - 1} {x\left[ {n + k} \right]} x\left[ n \right] $$

Equation 6 is defined as the biased estimator of the ACF and is usually preferred since it tends to have smaller mean square error (variance) and decays faster in finite datasets when compared with the unbiased estimate (with scaling term 1/(*N*-*k*) rather than 1/*N*), where *N* is the number of samples [14, 20, 22]. To estimate the coefficients and variance, the method first requires the estimation of the first model order AR process parameters (Eq. 7). This is then followed by a recursive implementation for obtaining successively higher model orders from *k* = 2 to the desired model order (Eqs. 8–10). In Eqs. 7–10 two subscript indices are used to easily identify the coefficients as *a*_*Order, Coef. Number*_ [20].

7 $$ a_{11} = - \frac{{R_{xx} \left[ 1 \right]}}{{R_{xx} \left[ 0 \right]}}\,\,\,,\,\,\,\,\sigma_{1}^{2} = \left( {1 - \left| {a_{kk} } \right|^{2} } \right)R_{xx} \left[ 0 \right] $$

8 $$ a_{kk} = \frac{{\left[ {R_{xx} \left[ k \right] + \sum\nolimits_{l = 1}^{k - 1} {a_{k - 1,l} R_{xx} \left[ {k - l} \right]} } \right]}}{{\sigma_{k - 1}^{2} }} $$

9 $$ a_{kr}\,=\, a_{k - r,r} + a_{kk} a_{k - 1,k - r}^{*} $$

10 $$ \sigma_{k}^{2} = \left( {1 - \left| {a_{kk} } \right|^{2} } \right)\sigma_{k - 1}^{2} $$

The Yule-Walker approach is computationally very efficient when the Levinson-Durbin algorithm is employed [14].

#### Covariance method

In the Covariance method, the data are windowed and the points within the interval are used to compute the variance of the white noise. The estimated autocorrelation function $c_{xx} \left[ {j,k} \right] = r_{xx} \left[ {j - k} \right]$ (summation of *N*-*p* lag products) for each window location *k* and the variance are calculated using the following equations [20–22]:

11 $$ c_{xx} \left[ {j,k} \right] = \frac{1}{N - P}\sum\limits_{n = p}^{N - 1} {x^{*} \left[ {n - j} \right]} x\left[ {n - k} \right] $$

12 $$ \hat{\sigma }^{2} = \rho_{MIN} = c_{xx} \left[ {0,0} \right] + \sum\limits_{k = 1}^{p} {a_{k} x_{n - k} } $$

#### Modified covariance method

In this method the AR parameters are estimated by minimizing the average (Eq. 13) of the estimated forward (Eq. 14) and backward (Eq. 15) prediction errors [21, 22].

13 $$ \hat{\rho } = \frac{1}{2}\left( {\hat{\rho }^{f} + \hat{\rho }^{b} } \right) $$

14 $$ \hat{\rho }^{f} = \frac{1}{N - P}\sum\limits_{n = p}^{N - 1} {\left| {x\left[ n \right] + \sum\limits_{k = 1}^{p} {a\left[ k \right]x[n - k]} } \right|}^{2} $$

15 $$ \,\hat{\rho }^{b} = \frac{1}{N - P}\sum\limits_{n = 0}^{N - 1 - p} {\left| {x\left[ n \right] + \sum\limits_{k = 1}^{p} {a^{*} \left[ k \right]x\left[ {n + k} \right]} } \right|}^{2} $$

The autocorrelation is estimated as

16 $$ c_{xx} \left[ {j,k} \right] = \frac{1}{{2\left( {n - p} \right)}}\left( {\sum\limits_{n = p}^{N - 1} {x^{*} } \left[ {n - j} \right]x\left[ {n - k} \right] + \sum\limits_{k = 0}^{N - 1 - p} {x[n + j]x^{*} \left[ {n + k} \right]} } \right) $$

#### Burg method

The Burg method computes the reflection coefficients directly (Eq. 17) and from these the remaining AR parameters are obtained using the Levinson-Durbin algorithm. The reflection coefficients are obtained by minimizing the average of the backwards and forwards prediction errors in a constrained manner when compared with modified covariance method. Burg’s method assumes that *a*_*kk*_ coefficient is estimated after the *a*_*kk*-1_ model order prediction error filter coefficients had been estimated by minimizing the *a*_*kk*-1_ model order prediction error power. First it is necessary to estimate the autocorrelation at lag zero with the forward and backward prediction errors (Eqs. 18 and 19). This is followed by the estimation of the reflection coefficients (Eq. 17) which are dependent of forward and backward prediction errors (Eqs. 11 and 12) [20–22]. $k = 1,{ 2},\, \ldots ,\,p .$

17 $$ a_{kk} = \frac{{ - 2\sum\nolimits_{n = k}^{N - 1} {\hat{e}_{k - 1}^{f} \left[ n \right]\hat{e}_{k - 1}^{b} \left[ {n - 1} \right]^{*} } }}{{\sum\nolimits_{n = k}^{N - 1} {\left( {\left| {\hat{e}_{k - 1}^{f} \left[ n \right]} \right|^{2} + \left| {\hat{e}_{k - 1}^{b} \left[ {n - 1} \right]} \right|^{2} } \right)} }} $$

The recursive estimation of the variance and coefficients for the higher model orders are calculated using Eqs. 8 and 9 (Levinson-Durbin algorithm).

18 $$ \hat{e}_{k}^{f} \left[ n \right] = \hat{e}_{k - 1}^{f} \left[ n \right] + a_{kk} \hat{e}_{k - 1}^{b} \left[ {n - 1} \right]\,\,\,\,\,\,\,n = k + 1,\,\,k + 2, \ldots ,N - 1 $$

19 $$ \hat{e}_{k}^{b} \left[ n \right] = \hat{e}_{k - 1}^{b} \left[ {n - 1} \right] + a_{kk}^{*} \hat{e}_{k - 1}^{f} \left[ n \right]\,\,\,\,\,\,\,\,n = k,\,\,k + 2,\, \ldots ,N - 1 $$

### Model order selection criteria

Since the AR model order is not known a priori, it is necessary to apply a model order estimation technique for finding the best model order for the AR model. In this study we used Criterion AR Transfer Function (CAT), a method suggested by Parzen [23], for identifying the AR model order. Equation 20 presents the CAT method where *p* is the optimum model order, $\sigma^{2}_{p}$ is the white noise variance and *N* is the number of samples of the data used.

20 $$ CAT_{p} = \left( {\frac{1}{N}\sum\limits_{j = 1}^{p} {\frac{N - j}{{N\sigma_{j}^{2} }}} } \right) - \left( {\frac{N - P}{{N\sigma_{p}^{2} }}} \right) $$

**Fig. 3:**
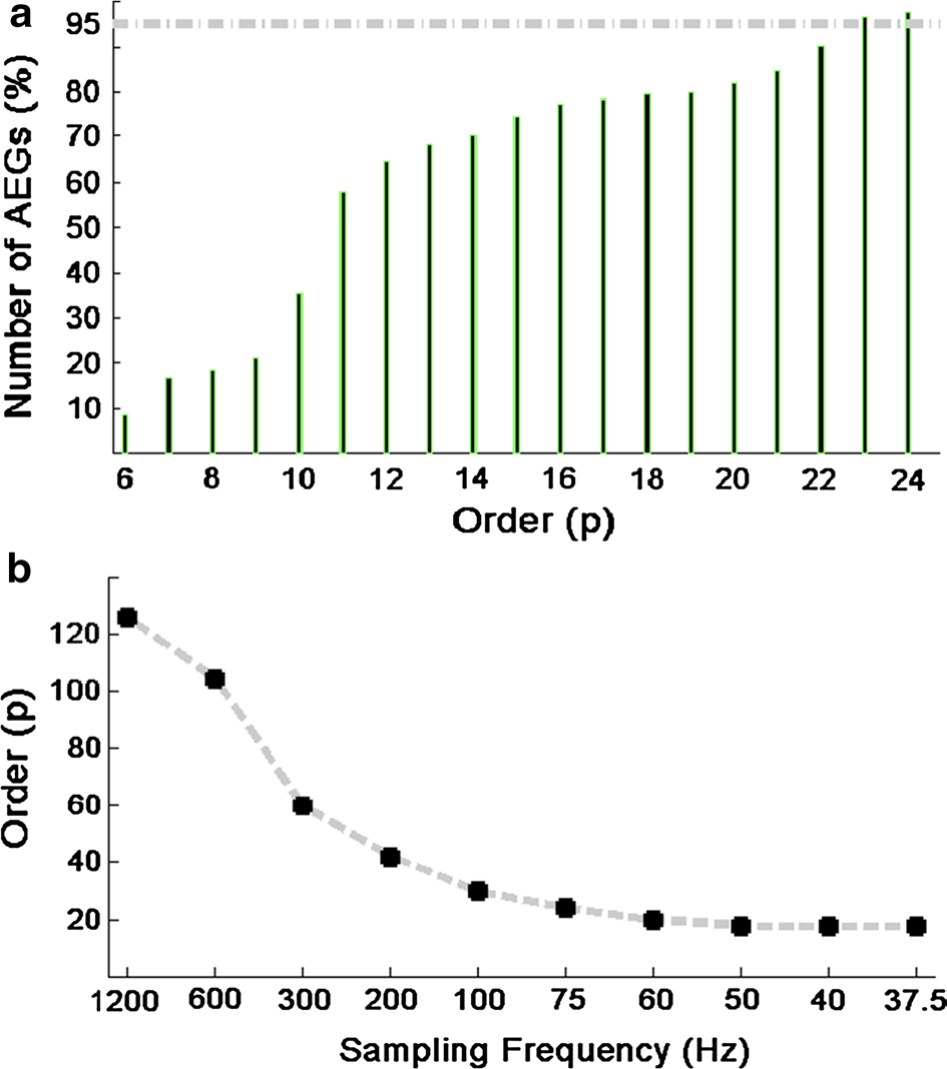
AR model order estimation. **a** Cumulative histogram for one patient showing percentage of AEGs (out of 2048) covered by the respective orders calculated by the CAT method (Fs = 75 Hz over 20.478 s). In this case p = 23 covers at least 95 % of all AEGs, but as odd orders were avoided (see text) we choose p = 24; **b** ‘Overall’ model order calculated for all patients using CAT technique for various Fs values. These overall order values chosen also cover at least 95 % of all AEGs (p = 126, 104, 60, 42, 30, 24; 20 and 18 respectively)

For each patient, the model order value for each of the 2048 AEGs, with segment length of 20.478 s and re-sampling frequency were estimated using CAT method. The model order value can be different for each AEG. Hence, a model order value that would mathematically attend the majority of the AEGs’ population is needed for generation of the 3D AR DF maps. The authors have defined this order, as the optimum order, identifying on the cumulative histogram the order that satisfies mathematically at least 95 % of the AEGs of the entire segment (illustrative example at Fig. 3a for one of the patients). Odd orders were avoided as one of their poles lies on the real axis so it does not affect much the DF; the order selected in this case is the next even value. As a next step, the model order value was extended for the remaining patients and different sampling frequencies strategies (please see Fig. 3b for illustration). The optimum model order values presented at Fig. 3b were obtained as above described by attending mathematically at least 95 % of 2048 AEGs with the segment length of 20.478 s for all patients.

### Statistical analysis

We fitted a linear mixed effects model to the data, which included as random effects: (1) patient, (2) the interaction patient and down sampling (DS) in the time domain of the AEGs with different sampling frequencies (down sampled signal) and (3) the interaction between patient, down sampled signal, and DF estimation using each of the AR spectral techniques (Yule-Walker, Covariance, Modified Covariance and Burg methods). As fixed effects we included: (1) DF estimation using each of the AR spectral techniques, (2) DS of the AEGs, and (3) the interaction between AR spectral estimation techniques and DS of the AEGs. These analyses tested whether any differences between the DF using the AR spectral estimation techniques varied by sampling frequency, while properly allowing for the nested structure of the data (i.e. where the DF calculated by each AR spectral technique was measured at each sampling frequency in each patient). The results were compared with those obtained using the FFT-based approach by the percentage of DF agreement. The analysis was performed using the *nlme* [24] package in *R* [25]. Mixed model ANOVA was used to study the effect of downsampling factor and DF estimation for the AR spectral techniques. P-values less than 0.05 were considered statically significant.

## Results

Eight male patients with symptomatic drug-refractory persAF were included in this study (mean age of 47 ± 4 years). Patients presented a history of persAF episodes of 34 ± 9 months with a moderated dilated LA (48 ± 2 mm) and left ventricle ejection fraction above 55 % (5 out of 8). Patient characteristics are summarized in Table 1 and represent largely what we might expect for a persAF population undergoing catheter ablation.

Figure 2 shows an AEG originally sampled at 1200 Hz with a total of 8192 samples (upper trace). A re-sampled signal with downsampling of 32 times (new Fs = 37.5 Hz) is shown on the second trace. Spectral analysis performed using FFT (for the original signal) and AR Yule-Walker (for the downsampled signal) illustrates that the DF of the signal can still be estimated after downsampling using the AR approach. Zero padding of 4 times resulted in a total of 32,768 samples produced a frequency step of 0.0366 Hz for the FFT approach. The PSD using AR Yule-Walker model was applied for two different AR model orders (50 and 18) and since the AR spectrum is continuous, the number of spectral samples was chosen so that frequency intervals were the same as applied by using the FFT approach using the original sampling frequency (Fs = 1200 Hz).

### Selection of model order

Model orders were estimated for different Fs and the results are illustrated in Fig. 3. Figure 3a shows the cumulative histogram of the AEGs (in  %) against model order for one patient whose original signals were downsampled to 75 Hz. The model order value chosen was 24. Figure 3b shows the average behaviour of the estimated best AR model order for all patients for different downsampling strategies. The model order values for each Fs are 126, 104, 60, 42, 30, 24; 20; 18. The curve shows that the order decreases with Fs, as expected. Lower model order values result in shorter processing time.

### Spectral analysis and 3D DF mapping

Three sequential 3D DF maps were generated with different Fs values and the results were concentrated on the impact of downsampling on the DF maps.

**Fig. 4:**
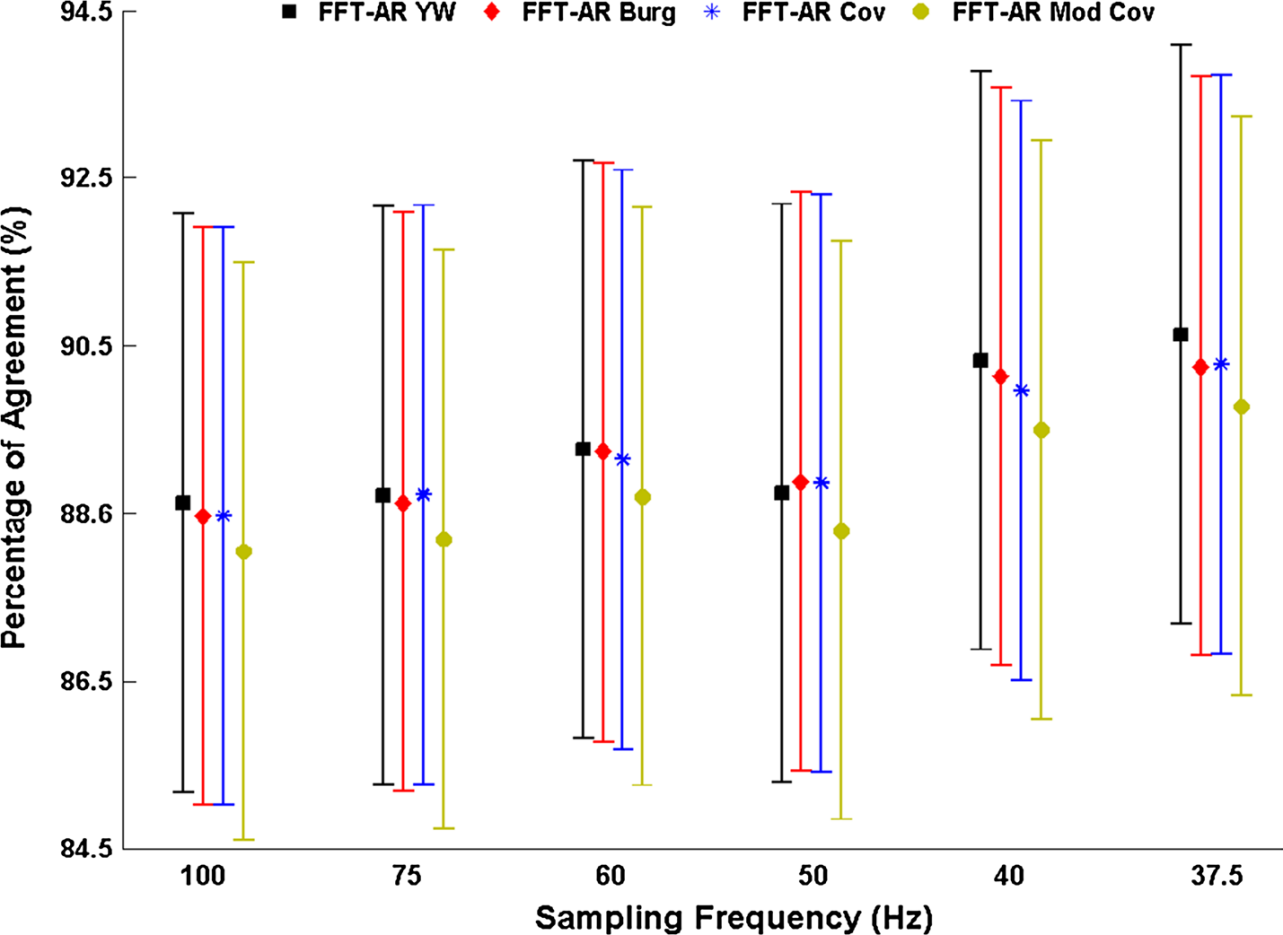
Percentage of agreement between the DF calculated using FFT and the DF calculated using the AR spectral analyses techniques with different re-sampling frequencies for the 2048 AEGs during 20.478 s (3 × 6.826 s). *Error bars* represent 95 % confidence intervals

For the AR spectral analysis, the mixed model ANOVA of the entire AF segment revealed that the interaction between downsampling factor and DF estimation with AR spectral techniques had a non-significant statistical effect (p = 1). In other words, our result showed that any differences between AR techniques did not depend on the re-sampling factor on the AEG. The main effect of the AR spectral estimation techniques (i.e. the effect of technique averaged over sampling frequency) was also not statistically significant (p = 0.62) as detailed in Fig. 4. This suggests no significant differences between AR techniques overall. Although there was no evidence of statistical differences between AR techniques, statistically significant differences between Fs values were found (p = 0.03). As Fig. 4 shows, Fs = 37.5 Hz had the greatest level of agreement, while the Fs = 100 Hz had the lowest level of agreement (albeit only around 2 % lower than at 37.5 Hz). DF values of all 2048 AEGs were compared between the 3D DF maps obtained by AR and FFT spectral analysis techniques. The total proportion of ‘similar’ DF points between techniques (percentage of DF agreement) is presented in Fig. 4.

**Fig. 5:**
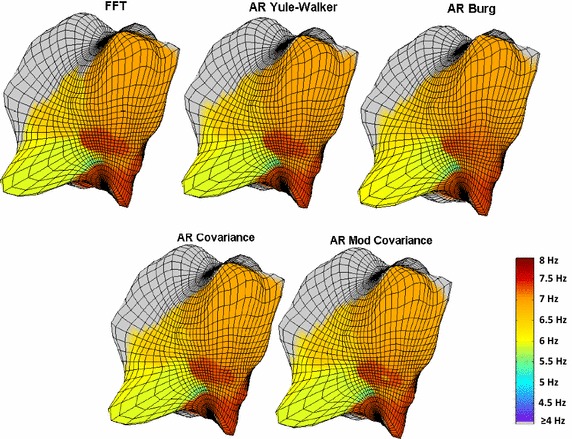
DF maps using different techniques for spectral estimation: FFT technique with the original Fs (1200 Hz); and four AR techniques. The AR methods used a re-sampled frequency of 37.5 Hz

Figure 5 shows a typical case of a 3D DF map using the FFT-based approach and the four AR techniques with the model order selection presented in Fig. 3b. Both methods result in similar 3D DF maps with a good agreement and the percentage of equal points between FFT vs. AR Yule-Walker was 93.8 %, FFT vs. AR Burg was 93.6 %, FFT vs. AR Covariance was 93.8 % and FFT vs. AR Modified Covariance was 93.2 %.

DF estimation using AR Yule-Walker method has the advantage of being computationally efficient. Aiming to observe the impact of the sampling frequencies to generate the 3D DF maps, the processing times of FFT and AR Yule-Walker were measured for estimation of 3D DF maps. The processing time for the FFT at Fs = 1200 Hz was 7.65 s and the processing time for the AR Yule-Walker, as expected, decreased with sampling frequency to: 5.44 s (Fs = 100 Hz), 5.35 s (Fs = 75 Hz), 5.32 s (Fs = 60 Hz), 5.27 s (Fs = 50 Hz), 5.15 s (Fs = 40 Hz) and 5.05 s (Fs = 37.5 Hz).

## Discussion

Spectral analysis has been used as a tool to provide information about the behaviour of the electrical activity of the heart. Areas with high frequency activity are thought to be ‘driving’ the rhythm in patients with AF and hence are possible targets for catheter ablation. 3D DF mapping using FFT-based approach has been shown to enable the location of high-frequency areas [26–28] and ablation of these areas seems to be an effective therapy in eliminating DF gradient and restoring sinus rhythm [26, 27]. The majority of the studies that identify DF of endocardial electrograms during EP procedures used FFT-based approaches. For time varying spectra usually short segments of the signal are used for spectral analysis and it is well known that the spectral resolution of Fourier-based spectral analysis is poor for shorter segments [12, 13]. Therefore, investigation of alternative spectral analysis methods to track DF using shorter time segments while maintaining good time-spectral resolution has its importance in the literature [13, 29–31]. In this study, we demonstrated that autoregressive spectral analysis can be used to generate dominant frequency maps of atrial electrograms of patients in persistent atrial fibrillation. The use of this technique in AF studies might be potentially avoided by reasons such as its time processing, the choice of the suitable AR technique and ‘model order’. The manuscript has covered all points by demonstrating that through a suitable downsampling strategy, AR could be performed faster than the FTT and with adequate model order selection. Moreover, apart from the technical ‘sophistication’ between the AR techniques to estimate the spectrum, the results were similar them and also similar with the FFT where all techniques were applied to electrograms with segment sizes commonly seen in AF studies.

For tracking the DF, two different definitions for DF are commonly presented in the literature [7, 32]. We have chosen the approach that has been used and validated previously with the St Jude´s system for noncontact AEGs [7]. In this approach, a high-pass filter is implemented and no further action is needed to identify the DF through spectral analysis after QRS-T removal [7, 19]. The AR spectral estimation techniques were implemented after a selection of appropriate sampling rate and AR model order to generate high-density 3D DF maps. As previously stated, our results demonstrated that the AR-based 3D DF maps produce good agreement when compared with the maps recently validated using FFT-based approach [15]. The agreement between AR and FFT techniques increased with higher levels of downsampling on the AEGs (Fig. 4). These results suggest that if downsampling of 32 times is implemented on AEG of persAF patients, the 3D DF maps of FFT and AR model will have good similarity (Fig. 5) with an average of the DF agreement at 93.6 ± 029 %. Although this result (better agreement with higher downsampling) might seem surprising, it is well-known that for AR-based spectral analysis the sampling rate should not be exaggerated compared to the Nyquist frequency [12].

It has been argued that Levinson-Durbin Yule-Walker produces poorer spectral resolution than other AR-based methods [14], however no statistically significant difference between Levinson-Durbin Yule-Walker and the other three techniques was observed. The advantage of the Levinson-Durbin Yule-Walker method is that it is faster than the other three techniques [14]. Although it is described in the literature differences of spectral estimation between the methods for test signals (with the Levinson-Durbin Yule-Walker method faring slightly worse than the other three approaches [22]), our results showed no statistical differences between the AR methods. Moreover, a slight advantage to Levinson-Durbin Yule-Walker in terms of DF similarity and processing time could be seen. This approach can substitute the frequency mapping systems currently used to help identify endocardium areas responsible for the AF maintenance and hence targets for ablation [7, 11, 15, 26, 27, 33].

We have tested several methods (not presented here) for estimation of AR model order and observed good agreement between them [34]. The CAT method was presented in this study. The model order values shown on Fig. 3 were implemented to generate the 3D AR DF maps. The effects of under/over estimating the order were explored by Schlindwein and Evans [34]. They showed that the effect on the spectral estimates of using too high a model order (twice the correct order for a known AR series) is usually not significant, while using too low an order (half the correct order for a known AR series) can change the estimate much more dramatically, that is, overestimating the AR model order is better than underestimating it. The comparisons of the spectral estimations obtained using the AR approach with the orders suggested with the FFT-based spectra show that no statistically significance overfitting was employed.

We have studied the three main issues that have made AR-based spectral analysis difficult for this application, namely the sampling frequency to be used, the AR model order to be chosen and the technique to find the AR coefficients. Our recommendations are that, for this application, the sampling frequency should be around 37.5 Hz, the order of the model should be 24 or slightly higher, and that the Yule-Walker Levinson-Durbin approach should be chosen to find the AR coefficients. We have shown that the AR-based DF maps of AEGs from persAF patients are very similar (more than 90 % of similarity) to those obtained using FFT-based maps. No statistically significant differences between the four AR techniques we tested were found, but AR Levinson-Durbin Yule-Walker has greater computational efficiency compared to the other three AR methods. Using the values and approach recommended above, the processing time (another issue that normally makes AR spectral estimation less attractive than the FFT-based approach) compares well with that of FFT, allowing for real-time implementation (AR-based spectral estimation takes 5.05 s for segments that are 6.826 s long).

Finally, concerning the limitations of the technology, AEGs acquired from remote areas (>4.0 cm) from the centre of the MEA have been shown to be significantly attenuated making the technology not suitable for large cardiac chambers. Movements of the balloon will produce distorted AEGs and as consequence, a new 3D geometry needs to be re-done if the balloon moves. The use of NCM to determine electrical activation in tubular structures (i.e. pulmonary vein, superior or inferior vena cava) is a challenge due to the “line-of-sight issue”.

## Conclusions

This study showed the feasibility of AR spectral estimation techniques for producing 3D DF maps with appropriate sampling rate and AR model order, offering an alternative approach for 3D DF computation in human persAF studies that might contribute as an auxiliary tool for the study of AF ablation.

## Abbreviations

AR: autoregressive
3D: three dimensional
DF: dominant frequency
AEGs: atrial electrograms
persAF: persistent atrial fibrillation
DS: downsampling
AF: atrial fibrillation
CFAE: complex fractionated atrial electrograms
FFT: fast fourier transform
PSD: power spectral density
LA: left atrium
NCM: noncontact mapping
MEA: multielectrode array catheter
EP: electrophysiological
Fs: sampling frequency
CAT: criterion AR transfer function
